# The Relationship between Motivational Climate and Personal Treatment Satisfaction among Young Soccer Players in Norway: The Moderating Role of Supportive Coach-Behaviour

**DOI:** 10.3390/sports8120162

**Published:** 2020-12-12

**Authors:** Tommy Haugen, Jan F. Riesen, Ketil Østrem, Rune Høigaard, Martin K. Erikstad

**Affiliations:** Department of Sport Science and Physical Education, University of Agder, 4604 Kristiansand, Norway; janriesen95@gmail.com (J.F.R.); ketil.ostrem@uia.no (K.Ø.); rune.hoigaard@uia.no (R.H.); martin.erikstad@uia.no (M.K.E.)

**Keywords:** athlete satisfaction, performance climate, mastery climate, coaching behaviour, moderation, soccer

## Abstract

Motivational climate and coach-behaviour seem important to understand sport involvement and participation. However, less is known about the potential interaction between these facets, and how it relates to athlete satisfaction. This study’s purpose is to examine the relationship between the perceived motivational climate, supportive coach-behaviour, and athletes’ personal treatment satisfaction among young soccer players. More specifically, we investigated the moderating effect of supportive coach-behaviour on the relationship between motivational climate and personal treatment satisfaction. Five hundred and thirty-two players (Mean age = 15.4 years, *SD* = 1.2) attending a Norwegian national soccer tournament participated in the study. Self-completion questionnaires were used to attain data. A linear regression analysis revealed that mastery of climate and supportive coach-behaviour were positively associated with personal treatment satisfaction. A negative association was found between performance climate and personal treatment satisfaction. Further, moderation analyses revealed that supportive coach-behaviour moderated the relationship between performance climate and personal treatment satisfaction. The findings indicate that a performance climate may not be as maladaptive when coaches provide supportive behaviour. The findings highlight the value of a further examination of the interaction between motivational climate and coaching behaviours, and its potential relations to young athlete’s sport experience.

## 1. Introduction

Numerous studies have shown beneficial outcomes for children and adolescents when participating in sports and physical activities [1,2,3]. What then becomes important is identifying what motivates children and adolescents to continue participating. Several researchers have highlighted social environmental factors and, notably, the relationship between coaches and athletes, to be important factors when understanding sport involvement and participation [4,5,6]. For instance, Molinero and colleagues [7,8] showed that one of the reasons for dropouts among youth was dislike of the coach. Similarly, coach programs which aimed to enhance athletes’ relationships with their coaches revealed reduced dropout rates, suggesting that athletes with higher satisfying values of their coaches (e.g., liking them more) stay longer in sport [9]. When Chelladurai and Riemer [10,11] classified various facets of athletes’ satisfaction, one such facet was satisfaction with personal treatment from the coach. The concept can be defined as “satisfaction with those coaching behaviours that directly affect the individual, yet indirectly affect team development” [11] (p. 141). Thus, personal treatment satisfaction includes athletes’ perceptions of their coaches’ ability to show empathy and treat the athlete well. Furthermore, it has been proposed that athletes who are satisfied with the relationship with their coaches, who are valued, respected, and trusted, are not only more likely to continue sport participation [12], but also more committed and successful [13,14]. Taken together, as athletes’ satisfaction regarding personal treatment from the coach may be a salient factor for reducing dropout and fostering continued sport participation, it may be considered interesting to investigate factors that may lead to athletes‘ satisfaction with personal treatment from the coach.

The motivational climate might be one of the potential aspects that could shed a light on what limits or enhances athlete satisfaction [15]. Central to the research on motivational climate is Achievement Goal Theory (AGT) [16]. When Ames [17] extended her research on AGT from educational to sport settings, she proposed that there were certain climates that reflected certain goal orientations (ego or task). Ames [17] differed between a ‘mastery’ and a ‘performance climate’. A mastery climate emphasises task and learning values, the mastery of skills, effort, and personal improvement in competence, whereas a performance climate emphasises superior competence to others, social comparison, and demonstration of superior ability.

Previous research has generally identified a mastery climate as more advantageous in sport settings. For example, a systematic review identified that mastery climate was positively associated with positive affective states (e.g., satisfaction), and performance climate to negative affective states (e.g., negative emotions) [18]. Several researchers have therefore advocated coaches to foster a mastery climate in sport settings [15]. However, it has been proposed that, given the competitive environment of sport, performance-oriented cues might be unavoidable [19]. Furthermore, Ommundsen and Roberts [19] suggested it might therefore be beneficial to couple a performance climate with mastery-oriented situational cues. According to them, mastery-oriented situational cues “… may help the athletes to better cope with the competitive element of sport as it may give them a broader basis for experiencing success” (p. 395).

What is of particular interest to this study is that sport psychologists have identified the coach and coaching behaviours as important features in the determination of how athletes perceive the motivational climate [15,17,20,21]. Ames [17] highlight that the behaviour from and the choices made by the coach contribute to conveying a certain motivational climate. In addition, the proposed notion by Ommundsen and Roberts [19] that mastery-oriented situational cues may moderate a performance climate, suggests that the coaches not only influence how athletes perceive the motivational climate, but also might help athletes to better cope with the competitive element of sport.

When Kristiansen and Roberts [22] examined how young elite athletes coped with the competitive and organizational demands of the Olympic sport environment, they reported that athletes relied on supportive coach-behaviour (e.g., social support) to help with the sporting environment. Furthermore, researchers have also found positive relationships between supportive coach-behaviours and personal treatment satisfaction. For example, findings have revealed positive relationships between social support and satisfaction with the coach, and sport experience among soccer players [23]. Moreover, when Riemer and Toon [24] examined the relationship between leadership (i.e., coaching) and satisfaction in tennis players, they found that social support, positive feedback, and democratic behaviour accounted for a significant amount of variance in personal treatment satisfaction. Coaches engaging in supportive behaviour tend to use positive reinforcement and encouragement rather than criticism and punitiveness [6].

Based on the literature reviewed above, it seems clear that the relationship between motivational climate, coach-behaviour, and athlete satisfaction is a complex and dynamic process. Indeed, Bronfenbrenner [25] claimed that “in ecological research, the principal main effects are likely to be interactions” (p. 38). Taken together, one could argue that investigating the potential interaction between supportive coach-behaviour and motivational climate might be relevant for understanding levels of personal treatment satisfaction among athletes. Thus, the purpose of the present study was twofold: (1) To investigate the predictive value of mastery climate (MC), performance climate (PC), and supportive coach-behaviour (SB) on athletes’ personal treatment satisfaction (PT); and (2) to investigate the moderating role of SB in the relationship between PC and PT, as well as between MC and PT. Based on the theoretical and empirical propositions outlined above, it was hypothesised that MC and SB positively predict PT, and PC negatively predicts PT. Further, we hypothesised that SB would moderate the predicted positive relationship between MC and PT, and that SB would moderate the predicted negative relationship between PC and PT.

## 2. Materials and Methods

### 2.1. Sample and Procedures

The sample for the present study was a part of a larger research project and has previously been used in [26]. A total of 532 players (167 females and 365 males) involved in a national youth soccer-tournament participated in the study. The 31% female participants mirrored the actual proportion of participants in the tournament. (The administration of the tournament reported 30% female and 60% male participants for the included age-cohorts.). Participants had an average age of 15.4 (*SD* = 1.2), had played soccer for a mean of 8.9 years (*SD* = 2.6), and participated in a mean of 2.8 (*SD* = 1.0) soccer practices per week. Head coaches of all age restricted (i.e., Under-15, Under-16, and Under-19) teams (*N* = 51) attending the tournament were contacted prior to the tournament and were informed about the purpose and procedure of the study. Thirty-nine teams (76%) agreed to participate. The questionnaires were filled out in a classroom setting, during the preliminary stages of the tournament. A test leader from the research group was present to inform the participant verbally and in writing about the purpose of the study, that information attained would be treated confidentially, that participation was anonymous and voluntary, and that filling out the survey was considered a consent of participation. The completed questionnaires were returned to the test leader present. The number of participants from each team varied from 8 to 23 players (*M* = 13.6, *SD* = 3.2). Ethical approval (ref:38393) was obtained from the Norwegian Social Science Data Services, and the procedures were in accordance with the ethical standards of the authors’ university.

### 2.2. Instruments

Personal treatment-satisfaction. The personal treatment-subscale from the Athlete Satisfaction Questionnaire (ASQ; [11]) was assessed, and contained five items (e.g., “The recognition I receive from my coach”, “The level of appreciation my coach shows when I do well”), and was measured through a 7-point Likert scale ranging from 1—“not at all satisfied” to 7—“extremely satisfied”. Higher scores reflect greater satisfaction. For preliminary analyses, principal component analyses (PCA) with varimax rotation (where appropriate) were used to examine the factor structure for all instruments. Because of the sufficient a-priori knowledge about which factors to extract, the present study used a fixed factor solution for all PCA’s. A PCA for the PT-subscale revealed one component with strong factor loadings (>0.50) on all items, and an eigenvalue above 1. Explained variance for the component was 70.3%, and Cronbach’s alpha for the subscale was 0.89, indicating good internal consistency [27].

Perceived Motivational Climate. To assess the motivational climate, the Norwegian version [19] of the Perceived Motivational Climate in Sport Questionnaire (PMCSQ; [28]) was used. The Norwegian version consists of 20 items. Each item was preceded by the stem “In this team…” and responses were measured using a 5-Point Likert scale from 1—“strongly disagree” to 5—“strongly agree”. Nine of the items aim to assess mastery climate (e.g., “each player’s improvement is important”), and eleven items aim to assess performance climate (e.g., “out-playing team-mates is important”). The PMSCQ has previously been used on other samples from the same population [29,30,31]. A PCA confirmed the two-component structure of the instrument. Two items had factor loadings <0.50 and were thus removed. A second analysis confirmed a two-component structure with mastery and performance climate as separate factors. Explained variance of Component 1 and Component 2 were 34.6% and 21.6%, respectively, and factor loadings were >0.50 for all remaining items. Each component had an eigenvalue above 1, and appropriate internal consistency (performance climate: *α* = 0.87; mastery climate: *α* = 0.93).

Supportive coach-behaviour. The SB-subscale from the Coaching Behaviour Questionnaire (CBQ; [32]) was used to assess supportive coach-behaviour. The subscale contained eight items (e.g., “My coach shows support for me even when I make a mistake”). Each item was responded by a 4-point Likert scale from 1—“strongly disagree” to 4—“strongly agree”, with higher scores reflecting greater supportive behaviour. A PCA revealed one component. One item had a factor loading <0.50 and was thus removed. The second PCA confirmed the one component structure. The eigenvalue was greater than 1 and accounted for 43.7% of the variance. All remaining items (7) had factor loadings >0.50. Cronbach’s alpha for the subscale was 0.78.

### 2.3. Statistical Analyses

All statistical analyses were conducted using IBM SPSS version 25 for Windows. Descriptive statistics are presented with Mean and Standard Deviation. Independent samples t-tests were performed to investigate potential gender differences in descriptives. Bivariate correlation analysis was used to investigate and establish relationships between PT, SB, MC, and PC, and linear regression analysis was used to test the predictive value of SB, MC, and PC on PT. PROCESS v3.4 [33] for SPSS was used to test the moderating effect of SB in the relationship between PC and PT, and MC and PT. This approach examines whether the effect of the focal predictor on an outcome variable is moderated by a third variable. Specific direct effects were tested at differing levels of the moderating variable; low (1 *SD* below the mean), at the mean, and high (1 *SD* above the mean). The interactions were tested in two separate analyses: Once with PC as the focal predictor and once with MC as the focal predictor. In addition, we used age, gender, and either MC or PC as covariates. Significance level was set at *p* < 0.05 for all analyses.

## 3. Results

### 3.1. Descriptive Statistics

Independent samples t-tests revealed statistically significant differences between male and female participants. On average, male participants were older (*M*(*SD*)_male_ = 15.5(1.2) vs. *M*(*SD*)_female_ = 15.1(1.3), *p* < 0.01), more experienced (*M*(*SD*)_male_ = 9.5(2.4) vs. *M*(*SD*)_female_ = 7.7(2.7), *p* < 0.01), engaged in more practices per week (*M*(*SD*)_male_ = 2.9(1.1) vs. *M*(*SD*)_female_ = 2.6(0.7), *p* < 0.01), and played more years for the same team (*M*(*SD*)_male_ = 6.5(3.7) vs. *M*(*SD*)_female_ = 4.8(3.6), *p* < 0.01) than their female counterparts.

### 3.2. Testing the Hypotheses

Table 1 shows bivariate correlations between central continuous variables. Results revealed statistically significant (*p* < 0.01) relationships between all variables, in the expected direction. Positive correlations were found between PT, SB, and MC. Negative correlations were found between PC and PT, SB, and MC.

The linear regression analysis is shown in Table 2. The regression model predicting PT was statistically significant (*R*^2^ = 0.374, *F* = 70.437, *p* < 0.01). 37.4% of the variance in PT was explained by the independent variables in this model. The model also revealed that all variables had a statistically significant relation to PT (*p* < 0.01). Both SB and MC were positively associated to PT with SB having a higher *β* value (0.512) than MC (*β* = 0.159). PC had a *β* = −0.126, and was thus negatively associated to PT.

**Figure 1:**
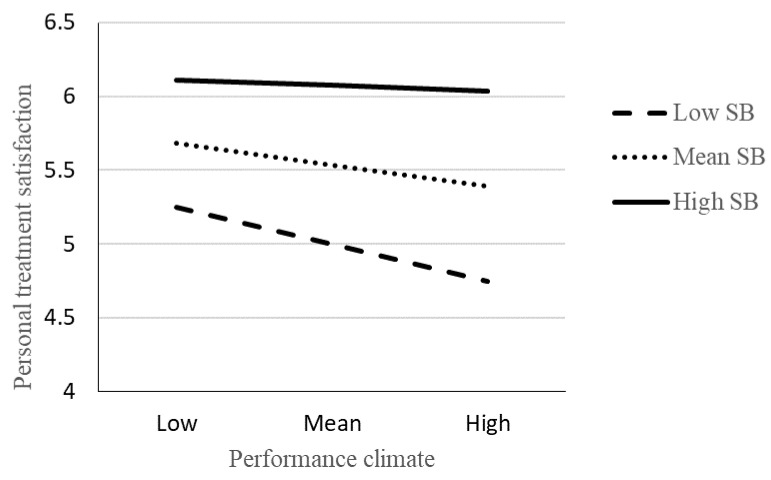
The relation between personal treatment satisfaction and performance climate moderated by supportive coach-behaviour. Note: SB = supportive coach-behaviour. Statistically significant interaction (*β* = 0.204, *t* = 2.347, *p* < 0.01). When SB was 1 *SD* below the mean, higher levels of PC predicted lower PT (*p* < 0.01). When SB was at the mean, higher levels of PC also predicted lower PT (*p* < 0.01). No significant negative prediction was shown when SB was 1 *SD* above the mean. Age and gender are included as covariates in the analysis.

**Figure 2:**
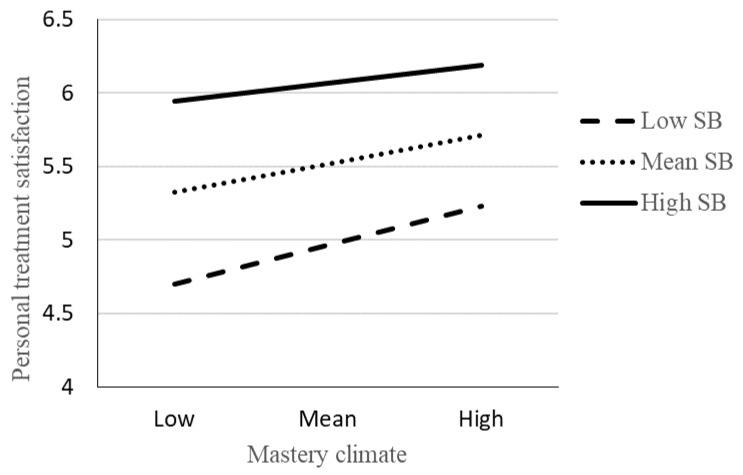
The relation between personal treatment satisfaction and mastery climate moderated by supportive coach-behaviour. Note: No statistically significant interaction (*β* = −0.129, *t* = −1.458, *p* = 0.14). Age and gender are included as covariates in the analysis.

Results of the moderation analyses are presented in Figure 1 and Figure 2. As can be seen, there was a statistically significant interaction effect of PC and SB on PT. When investigating the conditional effect of the predictor (PC) at values (Low SB: 1 *SD* below mean, Mean SB: Mean, and High SB: 1 *SD* above mean) of the moderating variable (SB), the results revealed a negative relationship between PC and PT for low SB (*β*(*se*) = 0.264(0.087), *p* < 0.01), a negative relationship between PC and PT for mean SB (*β*(*se*) = 0.187(0.053), *p* < 0.01), and a non-significant relationship between PC and PT for high SB ((*β*(*se*) = 0.111(0.062), *p* = 0.08). More specifically, this finding revealed that high levels of SB attenuated the negative relationship between PC and PT (Figure 1). No statistically significant interaction effect was found for MC and SB (Figure 2).

## 4. Discussion

In the present study, we examined the relationship between motivational climate, supportive coach-behaviour, and personal treatment satisfaction in youth soccer players. Our first objective was to investigate the predictive value of mastery climate, performance climate, and supportive coach-behaviour on young soccer players’ satisfaction with the personal treatment from the coach. Overall, results revealed that all variables had a statistically significant correlation. In sum, supportive coach-behaviour, mastery climate, and performance climate accounted for 37.4% of the variance in personal treatment satisfaction. This may indicate that supportive behaviour, mastery climate, and performance climate have a moderate [34] association with personal treatment satisfaction. Our results confirm previous research that emphasises the value of both situational aspects and coaching behaviours in the determination of athlete satisfaction [15,35].

In the present study, supportive coach-behaviour was positively associated with personal treatment satisfaction. This finding mirrors previous research [11], which noted that leadership (i.e., coaching) is a salient theme of personal treatment satisfaction. Our finding is also in accordance with previous research showing positive relationships between supportive behaviour (e.g., social support, positive feedback) and satisfaction with personal treatment [24].

Further, mastery climate was also positively associated with personal treatment satisfaction. Again, the finding was consistent with previous findings showing athletes perceiving the climate as mastery-oriented to respond more favourable in terms of satisfaction [36,37,38]. Moreover, it has been suggested that coaches should engage in behaviour that develops a sense of friendship and promotes trust and respect to foster a positive motivational climate [39]. Given that trust, respect, and appreciation are the very essence of satisfaction with personal treatment [13,14], one should expect to find positive relationships between mastery climate and personal treatment satisfaction, which we did.

Regarding our third hypothesized predictor, findings revealed that a perceived performance climate was negatively associated to personal treatment-satisfaction and were in accordance with similar research [36,37,38]. One explanation for this association may be due to the emphasis on normative rankings and comparisons in a performance climate. When coaches value normative ranking and comparison, they might focus much of their attention on high achieving athletes (more skilled, showing better performance), providing some athletes with greater support and feedback compared to others. Thus, some athletes may feel “overlooked”, ignored, and/or not appreciated, which in turn may negatively influence their personal treatment satisfaction.

In sum, the cross-sectional associations found in the present study advocate the theoretical notion for coaches to engage in supportive behaviour and foster a mastery climate to enhance athlete’s personal treatment satisfaction.

In order to gain a better understanding about the potential interaction between motivational climate and supportive coach-behaviour, our second study-objective was to examine the moderating role of supportive coach-behaviour in the relationship between performance climate and personal treatment satisfaction, and mastery climate and personal treatment satisfaction. We hypothesised first that the interaction of supportive behaviour and mastery climate would predict high levels of personal treatment satisfaction. This hypothesis was not supported in the present sample; the strength or direction of the relationship between mastery climate and personal treatment satisfaction was not conditional of levels of perceived supportive coach-behaviour. One possible explanation for this result may be that a mastery climate is independently important for facilitating satisfaction with personal treatment among athletes. Researchers have, for example, found that athletes perceived their coach to provide positive, informational, and encouraging feedback when they considered the climate to be mastery-oriented [40,41]. Thus, one may argue that when the athletes perceive the climate to be mastery-oriented, coaches already contribute to providing a positive and adaptive climate that improves personal treatment satisfaction, and that this positive association is not contingent on whether or not the coach (in addition) exhibits supportive behaviour. Our second hypothesis of moderation assumed that higher levels of supportive coach-behaviour would moderate the predicted negative association between perceived performance climate and personal treatment satisfaction. This hypothesis was supported in the present sample. For athletes scoring high (1 *SD* above sample-mean) on supportive coach-behaviour, no negative relationship between performance climate and personal treatment satisfaction was evident, whereas for athletes scoring mean or low (1 *SD* below sample-mean) on supportive coach-behaviour, the negative relationship was evident. This may indicate that perceptions of sufficient support from coaches might help athletes to better deal with the competitive and performance-oriented environment of sport (e.g., competition against others, rankings and normative evaluations) [42]. Athletes might feel that their coach trusts and values them, and thus attenuate the notion that one has to show superiority (e.g., superior skills and abilities) in order to receive recognition, even though the climate is perceived as performance-oriented. Thus, one may argue that our results supported and extended the notion proposed by [19], that a performance climate may not be (as) maladaptive when accompanied by adaptive situational cues.

All empirical studies have strengths and limitations. Particularly, some limitations must be considered when interpreting the results from the present study. One of the main issues with this study is the cross-sectional design. Establishing causal direction will therefore be impossible, since the design only allows us for a set of predictions based on theoretical assumptions [43]. In addition, several other factors could be potential moderators of the relationship between motivational climate and athlete satisfaction. Although investigating potential age- or gender-contingent relationships were beyond the scope of the present study, future research should consider exploring this further. Additionally, the present study has no information about the gender of the athletes’ coaches. Thus, the question remains whether athletes perceive female and male coaches’ behaviour differently. Moreover, we purely relied on self-reported data, and our results might be influenced by biased answers (e.g., “social desirability responding”). Lastly, our sampling method was based on a convenience approach. Because of that, one should be careful when extrapolating the results to other populations, because of the limited generalizability.

Future research would do well to focus on longitudinal study designs to determine the relationship between motivational climate, coach-behaviours, and athlete satisfaction over time. In addition, we encourage researchers to further examine the moderating effect of coaching behaviours in the relationship between the motivational climate and satisfaction (or other athlete outcomes). This approach would give us a better understanding for “whom” or under which conditions the effect occurs, and what strengthens or weakens the effect [44]. It would also be beneficial to investigate whether the investigated relations differ based on characteristics of the sport (e.g., individual sport vs. team sport).

## 5. Conclusions

The results from this study add further evidence to the role the motivational climate and coaching behaviours have on athlete outcomes. We believe that our results contribute to the existing literature and enhances our knowledge on motivational climate and coaching behaviour in relation to athlete satisfaction. Although not in a causal sense, our findings support the theoretical notion that coaches should foster a mastery climate and engage in supportive behaviour to enhance personal treatments satisfaction among youth sport athletes. Moreover, our findings also showed that high scores of supportive coach-behaviours mitigated the negative associations that perceived performance climate had on personal treatment satisfaction. Thus, we recommend that coaches should aim to building positive relationships with their athletes, in order to promote an environment that may foster positive experiences.

## Figures and Tables

**Table 1:** Bivariate correlations between central study variables.

	*M*	*SD*	PT	SB	MC	PC
PT	5.5	0.5	−			
SB	2.9	1.0	0.566 ^**^	−		
MC	3.6	0.9	0.271 ^**^	0.174 ^**^	−	
PC	2.6	0.8	−0.299 ^**^	−0.267 ^**^	−0.192 ^**^	−

Note. PT = personal treatment-satisfaction, SB = supportive coach-behaviour, MC = mastery climate, PC = performance climate, *M* = mean, *SD* = standard deviation. ^**^ *p* < 0.01.

**Table 2:** Linear regression, predicting personal treatment-satisfaction.

		*Β*	*t*	*R* ^2^
				0.374
Const.	2.453		6.646	
PC		−0.126 ^**^	−2.797	
MC		0.159 ^**^	3.632	
SB		0.512 ^**^	11.460	

Note. SB = supportive coach-behaviour, MC = mastery climate, PC = performance climate, *B* = standardized coefficient (beta), *R^2^* = adjusted R square. ** *p* < 0.01.
